# Impact of Handgrip Strength on Survival in Hemodialysis Patients

**DOI:** 10.3390/diagnostics15010075

**Published:** 2024-12-31

**Authors:** Kyungho Park, Seongyeop Jeong, Hyerim Park, Eu Jin Lee, Young Rok Ham, Ki Ryang Na, Dae Eun Choi

**Affiliations:** 1 Department of Nephrology, Chungnam National University Hospital, Daejeon 35015, Republic of Korea; ds3ixc@gmail.com (K.P.); surejojo123@daum.net (S.J.); eujinlee@cnuh.co.kr (E.J.L.); youngrok01@cnuh.co.kr (Y.R.H.); drngr@cnu.ac.kr (K.R.N.); 2 Department of Medical Science, Medical School, Chungnam National University, Daejeon 35015, Republic of Korea; hye05240@gmail.com

**Keywords:** hemodialysis, handgrip strength, muscle mass, bioimpedance spectroscopy, survival

## Abstract

**Background**: Hemodialysis patients face a high mortality risk, requiring effective clinical assessments. In these patients, muscle wasting due to protein-energy wasting (PEW) leads to increased frailty, which is strongly associated with worse outcomes, including higher mortality. As muscle mass declines, so does functional capacity, making regular assessment of both muscle mass and function critical for prognostic evaluation. Handgrip strength (HGS) offers a quick and reliable measure of muscle strength and functional capacity. In this study, we focused on the impact of HGS on survival in hemodialysis patients, analyzing its relationship with muscle mass and BMI. **Methods**: This retrospective cohort study included 408 dialysis patients (221 males, 187 females) who underwent bioimpedance spectroscopy (BIS) and HGS assessments between March 2021 and August 2023. Data collected included BIS profiles, HGS, dialysis status, age, complete blood count, blood chemistry, mortality, and CONUT scores. **Results**: Cox proportional hazards regression analysis revealed that lean tissue index (LTI) (HR 3.30, 95% CI 1.75–6.19), body mass index (BMI) (HR 2.65, 95% CI 1.17–6.01), and handgrip strength (HGS) (HR 4.22, 95% CI 2.05–8.70) were significant predictors of survival in the overall dialysis patient cohort. Gender-specific analysis showed that in males, both LTI (HR 4.81, 95% CI 1.89–12.23) and HGS (HR 5.45, 95% CI 2.18–13.61) significantly predicted survival. In females, HGS (HR 6.01, 95% CI 2.42–14.94) was a significant predictor, while LTI was also significant (HR 3.22, 95% CI 1.24–8.40, *p* = 0.017). In the multivariate Cox proportional hazards analysis, which adjusted for age, diabetes mellitus (DM), hypertension (HTN), BMI, fat tissue index (FTI), LTI, serum albumin, C-reactive protein (CRP), and CONUT score, HGS remained a significant predictor of survival in female dialysis patients (HR 2.77, 95% CI 1.00–7.65, *p* = 0.049). **Conclusions**: HGS has been identified as an important factor for survival in dialysis patients, particularly in female patients, independent of muscle mass and BMI.

## 1. Introduction

Patients undergoing hemodialysis have a significantly higher mortality rate compared to the general population [1], with protein energy wasting (PEW) and increased vulnerability to sarcopenia being known as major contributing factors [2,3]. These patients often face numerous complications related to malnutrition, inflammation, and reduced physical function, all of which compound their risk of adverse outcomes [4]. Effective tools for assessing patient risk are crucial to improving clinical outcomes and guiding timely interventions.

Sarcopenia, as defined by the European Working Group on Sarcopenia In Older People 2, is characterized by both a decline in muscle mass and muscle strength [5]. However, recent evidence suggests that low muscle strength is more strongly associated with aging, protein-energy wasting (PEW), physical inactivity, inflammation, and mortality compared to low muscle mass alone [6]. This highlights the limitations of assessing muscle mass in isolation and underscores the added diagnostic and prognostic value of evaluating muscle strength.

Importantly, muscle strength is not solely dependent on muscle mass, and the relationship between the two is not always direct. As people age, muscle strength tends to decline more rapidly than muscle mass [7], and in some cases strength can decrease even when muscle mass is preserved or increased [8]. Therefore, muscle strength and muscle mass should be considered distinct entities, each with unique clinical implications. Given these differences, the assessment of muscle strength may provide more valuable insights into the health and survival of patients, particularly in the context of conditions like sarcopenia and PEW.

Handgrip strength (HGS) has emerged as a simple, non-invasive measurement that correlates well with nutritional status, muscle mass, inflammation level, functional capacity, and is equally applicable to hemodialysis patients [9,10,11,12]. Several studies have indicated that lower HGS is strongly associated with increased mortality risk, making it a potentially valuable predictor of survival [13,14,15,16]. However, the interaction between HGS and other risk factors such as body mass index (BMI), body composition, and comorbidities like diabetes and hypertension remains insufficiently explored.

This study aimed to evaluate the prognostic value of HGS in predicting survival in hemodialysis patients and its relationship with muscle mass and BMI to improve risk stratification and patient management.

## 2. Materials and Methods

### 2.1. Study Design

This study was a single-center retrospective cohort conducted at Chungnam National University Hospital, Daejeon, Republic of Korea. We reviewed the medical records of patients who underwent hemodialysis and had HGS and bioimpedance spectroscopy (BIS) measurements between January 2021 and August 2023. Laboratory results were also collected from the medical records. For patients who had their BIS measured more than twice during the study period, only the first measurement was used for analysis. Patients with incomplete pre-dialysis BIS results, incomplete laboratory data, or incomplete HGS data were excluded from the study. The study protocol was reviewed and approved by the Institutional Review Board of Chungnam National University Hospital (IRB No. 2023-11-0841)

### 2.2. Assessment of Body Composition and HGS

Body composition was assessed using the Body Composition Monitor (Fresenius Medical Care, Bad Homburg, Germany), a portable bioimpedance spectroscopy device. All measurements were performed by a trained nurse with the patients in a relaxed supine position for at least 5 min prior to measurement. BIS measurements were taken before dialysis. The data collected included overhydration (OH), total body water (TBW), extracellular water (ECW), intracellular water (ICW), lean tissue index (LTI), fat tissue index (FTI), lean tissue mass (LTM), fat mass, body cell mass (BCM), and dry weight.

HGS was measured using the Takei T.K.K.5401 GRIP-D handgrip dynamometer (Takei Scientific Instruments Co., Ltd., Tokyo, Japan). Measurements were taken after a 5-min rest period using the non-fistula arm. HGS was assessed concurrently with BIS measurements, following instructions provided by a trained nurse.

### 2.3. Assessment of Clinical Parameters and Outcome Measurement

Information on the patients’ baseline characteristics and comorbidities was collected by reviewing previous medical records. Major comorbidities included diabetes and hypertension. The primary outcome was all-cause mortality, identified through hospital record review. Cox proportional hazards regression, Kaplan–Meier survival analysis, and ROC analysis were performed to evaluate the predictive value of HGS for mortality.

### 2.4. Statistical Analysis

Continuous variables were tested for normality using the Shapiro–Wilk test. Normally distributed variables are expressed as mean ± standard deviation and were compared using the independent *t*-test, while non-normally distributed variables are expressed as median (interquartile range) and were compared using the Mann–Whitney U test. Categorical variables are presented as N (%), and the Chi-square test was used for comparisons. A *p*-value of <0.05 was considered statistically significant. ROC analysis was used to evaluate the predictive value of LTI, FTI, BMI, and HGS for estimating all-cause mortality. Youden’s index was used to select the optimal cut-off point for each variable, and this analysis was applied separately to the entire cohort, as well as male and female groups (Figure S1 and Table S1). Based on these cut-off values, LTI, FTI, BMI, and HGS were categorized into low and high groups. Survival rates between the low and high groups, categorized based on the cut-off values of LTI, FTI, BMI, and HGS, were analyzed using Cox proportional hazards analysis. For HGS, multivariate Cox proportional hazards analysis was conducted to adjust for various confounding factors, including age, diabetes mellitus (DM), hypertension (HTN), BMI, FTI, LTI, serum albumin, C-reactive protein (CRP), and CONUT score. Kaplan–Meier curves were plotted to compare survival times between patients classified by HGS group (high vs. low). All statistical analyses were performed using R (version 4.4.0, R Foundation for Statistical Computing, Vienna, Austria).

## 3. Results

### 3.1. Baseline Characteristics

Between 1 January 2021 and 31 August 2023, a total of 575 patients underwent HGS and BIS measurements. Of these, 167 patients were excluded due to incomplete data, resulting in a final cohort of 408 patients for analysis. The median follow-up period for the dialysis group was 1.83 years, during which 39 patients in the dialysis group died.

The optimal HGS cut-off values for predicting all-cause mortality were determined using Youden’s index for the entire population, as well as for male and female patients separately. The optimal cut-off values were 18.4 for the overall population, 10.15 for female patients, and 18.45 for male patients. Based on these values, patients were divided into high HGS and low HGS groups.

Among female patients (*n* = 187), those in the high HGS group were significantly younger than those in the low HGS group [64.0 (16.5) vs. 78.0 (13.8) years, *p* < 0.001], and had a significantly longer survival period [23.7 (14.7) vs. 12.2 (14.3) months, *p* < 0.001]. In addition, the mortality rate was lower in the high HGS group compared with the low HGS group (7.3% vs. 25.0%, *p* = 0.005). There were no significant differences in the prevalence of hypertension or diabetes mellitus. The high HGS group had greater height (153.7 ± 6.4 vs. 149.5 ± 6.1 cm, *p* < 0.001) and higher body weight [56.5 (13.8) vs. 53.5 (12.3) kg, *p* = 0.024]. While systolic blood pressure (BP) did not differ significantly, diastolic BP was higher in the high HGS group [73.0 (19.0) vs. 68.0 (11.2) mmHg, *p* = 0.027]. There were no significant differences in BMI, lean tissue index, or fat tissue index between the two groups. Hemoglobin, albumin, blood urea nitrogen (BUN), total cholesterol, and C-reactive protein (CRP) levels were also comparable. However, serum creatinine concentrations were significantly higher in the high HGS group [6.9 (4.5) vs. 4.4 (3.1) mg/dL, *p* = 0.003]. Nutritional status assessed by the CONUT score indicated a better profile in the high HGS group [2.0 (3.0) vs. 3.5 (2.2), *p* = 0.037] (Table 1).

In male patients (*n* = 221), the high HGS group likewise showed a younger age [65.0 (12.0) vs. 76.5 (11.5) years, *p* < 0.001] and a longer survival period [24.1 (14.4) vs. 14.5 (16.5) months, *p* < 0.001]. The mortality rate was also lower in the high HGS group (4.8% vs. 20.4%, *p* = 0.001). No significant differences were observed in the prevalence of hypertension or diabetes mellitus. Height [167.0 (8.0) vs. 162.2 (7.0) cm, *p* < 0.001], body weight [70.6 (14.7) vs. 62.8 (11.6) kg, *p* < 0.001], and BMI [25.5 (4.4) vs. 23.6 (3.5) kg/m^2^, *p* = 0.001] were all significantly higher in the high HGS group. Diastolic BP was also higher in the high HGS group [76.0 (22.5) vs. 64.5 (12.0) mmHg, *p* < 0.001]. In body composition analysis, the high HGS group exhibited a higher lean tissue index [13.5 (3.4) vs. 11.9 (4.5) kg/m^2^, *p* = 0.001], while fat tissue index did not differ. The high HGS group had significantly higher hemoglobin and albumin levels, as well as BUN and serum creatinine. CRP levels were lower in the high HGS group [0.1 (0.7) vs. 0.5 (1.0) mg/L, *p* = 0.006]. The CONUT score was also significantly lower in the high HGS group [3.0 (2.0) vs. 4.0 (2.8), *p* = 0.004], indicating better nutritional status (Table 1).

### 3.2. Comparative Cox Proportional Hazards Regression Analysis of LTI, FTI, BMI, and HGS on Survival in Dialysis Patients

To assess the impact of lean tissue index (LTI), fat tissue index (FTI), body mass index (BMI), and handgrip strength (HGS) on survival in dialysis patients, Cox proportional hazards regression analyses were performed. The findings are summarized in Table 2.

**Figure 1:**
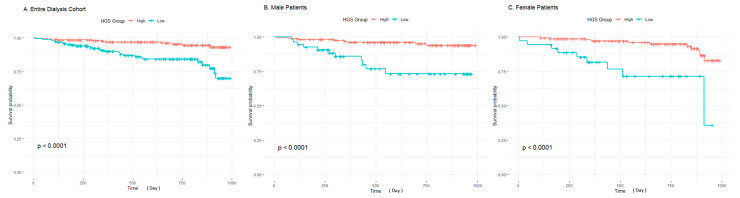
Kaplan–Meier curves comparing survival rates among hemodialysis patient groups stratified by HGS: (**A**) shows survival rates for the entire hemodialysis cohort, with a clear distinction in survival between patients with high and low HGS. (**B**) depicts survival rates in male patients, where low HGS is significantly associated with reduced survival compared to high HGS. (**C**) demonstrates survival rates in female patients, similarly showing that low HGS correlates with worse survival outcomes. Across all groups, a significant difference in survival based on HGS is evident, highlighting the prognostic value of handgrip strength in both males and females. The *x*-axis represents the survival period (in days), and the *y*-axis represents the survival probability.

In the overall cohort, low LTI was significantly associated with an increased risk of mortality (HR 3.30, 90% CI: 1.75–6.19, *p* < 0.001), whereas FTI did not show a significant association with mortality (HR 1.55, 90% CI: 0.76–3.18, *p* = 0.231). BMI, however, emerged as a significant predictor of mortality (HR 2.65, 90% CI: 1.17–6.01, *p* = 0.020). Lower HGS was strongly linked to a higher risk of mortality (HR 4.22, 90% CI: 2.05–8.70, *p* < 0.001), as further visualized in the Kaplan–Meier survival curves (Figure 1). Figure 1 illustrates survival probabilities stratified by high and low HGS groups, with Panel A depicting the entire dialysis cohort, while Panels B and C represent gender-specific analyses for male and female patients, respectively. Across all groups, lower HGS was associated with significantly reduced survival.

When analyzed by gender, male patients with low LTI were found to have a significantly higher risk of mortality (HR 4.81, 90% CI: 1.89–12.23, *p* < 0.001), while FTI and BMI did not show statistical significance in predicting mortality in this group. However, HGS remained a robust predictor of mortality among male patients (HR 5.45, 90% CI: 2.18–13.62, *p* < 0.001), as shown in Figure 1B. In female patients, low LTI was similarly associated with increased mortality (HR 3.22, 90% CI: 1.24–8.40, *p* = 0.017), while FTI and BMI did not exhibit statistically significant associations. Like in males, lower HGS was significantly associated with increased mortality in females (HR 6.01, 90% CI: 2.42–14.94, *p* < 0.001), as shown in Figure 1C.

### 3.3. Association of Handgrip Strength with Mortality: Multivariate Cox Proportional Hazards Models with Adjustment for Confounders

Further analysis was conducted to assess the association between HGS and mortality, using three different Cox proportional hazards models, as shown in Table 3. Model 1 was unadjusted, Model 2 was adjusted for key confounders (age, diabetes mellitus (DM), hypertension (HTN), BMI, FTI, and LTI), and Model 3 included additional adjustments for nutritional and inflammatory markers (serum albumin, C-reactive protein (CRP), and the Controlling Nutritional Status (CONUT) score).

In the univariate analysis (Model 1), lower HGS was strongly associated with increased mortality in dialysis patients (HR 4.22, 90% CI: 2.05–8.70, *p* < 0.001). This association remained significant after adjusting for age, DM, HTN, BMI, FTI, and LTI in Model 2 (HR 3.36, 90% CI: 1.54–7.34, *p* = 0.002). With further adjustments for nutritional and inflammatory factors in Model 3, the association was slightly attenuated but remained statistically significant (HR 2.86, 90% CI: 1.30–6.28, *p* = 0.009).

In male patients, low HGS was a significant predictor of mortality in the unadjusted Model 1 (HR 5.45, 90% CI: 2.18–13.62, *p* < 0.001) and continued to be significant in Model 2 (HR 4.48, 90% CI: 1.63–12.28, *p* = 0.004). However, after adjusting for nutritional and inflammatory markers in Model 3, the association between HGS and mortality in males was no longer statistically significant (HR 2.59, 90% CI: 0.83–8.11, *p* = 0.102).

In female patients, lower HGS was significantly associated with increased mortality across all models. In the unadjusted Model 1, the HR was 6.01 (90% CI: 2.42–14.94, *p* < 0.001). After adjusting for age, DM, HTN, BMI, FTI, and LTI in Model 2, the association remained strong (HR 3.50, 90% CI: 1.28–9.57, *p* = 0.015). Even after additional adjustments for nutritional and inflammatory markers in Model 3, the association between HGS and mortality remained statistically significant (HR 2.77, 90% CI: 1.00–7.65, *p* = 0.049).

## 4. Discussion

In this study, we highlighted the critical role of HGS in predicting survival among hemodialysis patients, alongside other key body composition metrics such as LTI, FTI, and BMI. Our findings demonstrate that HGS, LTI, and BMI are important predictors of survival across the dialysis population.

Previous studies have consistently supported the prognostic value of HGS in dialysis patients. B. P. Vogt et al. identified HGS as an independent predictor of all-cause mortality in dialysis patients, with cut-off values of 22.5 kg for men and 7 kg for women, even after adjusting for demographic, clinical, and nutritional factors [17]. Similarly, Matos et al. confirmed that low HGS is associated with a more than twofold increased risk of mortality in both male and female patients, with a stronger impact observed in women after considering nutritional status [18].

HGS’s role as a strong predictor of survival can be attributed to its association with functional status, muscle mass, and nutritional health [19,20]. Unlike other body composition parameters, HGS captures the functional component of muscle strength, which directly impacts patients’ ability to perform daily activities [21]. This in turn influences their quality of life and resilience to clinical complications. Notably, the robust predictive power of HGS, even when controlling for other variables like BMI and LTI, highlights that muscle function provides a unique prognostic value, beyond what can be inferred from muscle mass alone. This emphasizes that in dialysis patients, muscle function is even more important than muscle quantity in assessing overall health.

In this study, HGS emerged as a stronger predictor of survival in female hemodialysis patients compared to males, whereas LTI was more significant in predicting survival in males. These findings suggest that muscle function and body composition may impact survival differently depending on gender, a pattern observed in prior studies.

In studies conducted on the general elderly population, García-Hermoso et al. found that higher HGS was associated with reduced all-cause mortality in both men and women, but the protective effect was more pronounced in women [22]. Similarly, Ryuichi Kawamoto et al. showed that in men, both muscle strength (HGS) and muscle mass (thigh circumference) were key predictors of mortality, while in women, only HGS had a significant association with survival [23]. These studies collectively suggest that while muscle mass may be more relevant to survival in men, muscle function plays a critical role in both genders, especially in women.

Through this study, we confirmed that HGS was a stronger predictor of survival in women, while LTI was more significant in men among dialysis patients. Although there is limited research specifically addressing these gender differences in the dialysis population, our findings highlight the need for further investigation into gender-specific approaches when assessing muscle health and survival in dialysis patients.

An unexpected finding in this study was that, although BMI showed a significant association with survival when all patients were analyzed together, it did not have a significant impact on survival when analyzed separately by gender. In dialysis patients, unlike in the general population, a higher BMI is associated with better survival, a phenomenon known as the “obesity paradox” [24]. This paradox is often attributed to the reverse epidemiology observed in maintenance dialysis patients, where cardiovascular risk factors such as obesity, hypercholesterolemia, and hypertension act as protective factors, contrary to their effects in the general population. This unusual pattern may be related to the malnutrition-inflammation complex syndrome, which results from the combined effects of malnutrition and inflammation [25].

Stenvinkel et al. suggested that inflammation is a key factor explaining this paradox. While weight loss may benefit obese patients without inflammation, it may be detrimental to those with inflammation. In patients with elevated CRP levels (>10 mg/L) or low albumin levels (<35 g/L), obesity was found to have a protective effect on survival [26].

However, while BMI is a simple metric for assessing obesity, it does not distinguish between fat mass and lean body mass, which is a significant limitation. Noori et al. reported that higher fat mass (FM) in both sexes and higher lean body mass (LBM) in women appear to be protective, with the survival advantage of FM being superior to that of LBM [27]. In contrast, our study found that LTI had a significant impact on survival, whereas FTI did not. In Noori et al.’s study, FM and LBM were measured using near-infrared interactance technology, whereas our study utilized bioimpedance spectroscopy (BIS), which, by accounting for fluid overload in dialysis patients, provides a more accurate assessment of muscle mass. BIS applies a three-compartment model and has been shown to be as reliable as dual-energy X-ray absorptiometry (DXA) for body composition evaluation in patients with chronic kidney disease [28,29,30].

Our study further clarifies the obesity paradox by demonstrating that LTI is more clinically relevant to survival outcomes than BMI or FTI, particularly in male patients. This highlights the importance of incorporating body composition assessments that differentiate between lean and fat tissue for accurate risk stratification and management. BIS provides a detailed understanding of muscle and fat distribution, which is especially relevant in male patients, given the strong association between LTI and survival outcomes. In contrast, HGS serves as a stronger predictive marker for survival in female patients, emphasizing the critical role of muscle function over muscle quantity in this subgroup. Therefore, routine evaluation of both muscle function and composition should be considered to improve survival outcomes in dialysis patients, with a focus on gender-specific approaches.

One of the key strengths of this study is the use of BIS, which allows for a more precise evaluation of body composition, particularly in accounting for fluid overload, a common issue in dialysis patients. BIS’s accuracy, comparable to that of DXA, enhances the reliability of our findings regarding the relationship between LTI, FTI, and survival. Additionally, our study provides important gender-specific insights, demonstrating the differential impact of HGS and body composition on survival in male and female dialysis patients, an area that has been underexplored in previous research.

However, this study is not without limitations. First, its retrospective nature may introduce selection bias, and the relatively small sample size could limit the generalizability of the findings. Second, although we controlled for key confounders, residual confounding due to unmeasured variables (e.g., physical activity levels, comorbid conditions) and the inability to include kt/V values for all patients represent limitations of our study. Collecting kt/V data was challenging, as many patients received regular dialysis treatments at external dialysis facilities. kt/V data were available for less than 50% of the total patient population, and, furthermore, the measurements were often taken at time points significantly distant from the BIS assessment, making them unsuitable for reliable analysis. Consequently, the exclusion of kt/V from the confounder analysis represents a limitation of this study. Third, our study included a relatively large number of covariates in the multivariable Cox regression analysis relative to the number of events, which may have increased the risk of overfitting. This limitation could potentially affect the robustness and generalizability of the findings. Future studies with larger sample sizes and event numbers are warranted to validate these results. Lastly, the single-center design limits external validity, and further studies are needed to confirm these findings in diverse dialysis populations.

## 5. Conclusions

HGS is a robust and independent predictor of survival in hemodialysis patients, particularly among females. Our findings suggest that incorporating HGS measurement into routine clinical assessments may enhance risk stratification and improve patient management.

## Figures and Tables

**Table 1:** Baseline characteristics of hemodialysis patients stratified by handgrip strength (HGS) in female and male subgroups: continuous variables are expressed as mean ± standard deviation for normally distributed data, and as median (interquartile range) for non-normally distributed data. Categorical variables are presented as N (%).

	Women (*n* = 187)			Men (*n* = 221)		
	High HGS *n* = 151	Low HGS *n* = 36	*p*-Value	High HGS *n* = 167	Low HGS *n* = 54	*p*-Value
Age (years)	64.0 (16.5)	78.0 (13.8)	<0.001	65.0 (12.0)	76.5 (11.5)	<0.001
Survival period (month)	23.7 (14.7)	12.2 (14.3)	<0.001	24.1 (14.4)	14.5 (16.5)	<0.001
Number of deaths (%)	11 (7.3%)	9 (25.0%)	0.005	8 (4.8%)	11 (20.4%)	0.001
Hypertension	96 (63.6%)	22 (61.1%)	0.934	119 (71.3%)	40 (74.1%)	0.821
Diabetes mellitus	75 (49.7%)	21 (58.3%)	0.454	112 (67.1%)	35 (64.8%)	0.89
Height (cm)	153.7 ± 6.4	149.5 ± 6.1	<0.001	167.0 (8.0)	162.2 (7.0)	<0.001
Weight (kg)	56.5 (13.8)	53.5 (12.3)	0.024	70.6 (14.7)	62.8 (11.6)	<0.001
Systolic BP (mmHg)	145.7 ± 25.3	143.7 ± 22.7	0.656	148.9 ± 24.7	143.3 ± 24.6	0.154
Diastolic BP (mmHg)	73.0 (19.0)	68.0 (11.2)	0.027	76.0 (22.5)	64.5 (12.0)	<0.001
BMI (kg/m^2^)	23.7 (5.6)	23.8 (4.3)	0.507	25.5 (4.4)	23.6 (3.5)	0.001
Overhydration (L)	1.2 (1.9)	0.5 (2.1)	0.094	2.0 (2.5)	1.6 (2.3)	0.454
Lean tissue index (LTI) (kg/m^2^)	10.8 (2.6)	9.8 (2.9)	0.173	13.5 (3.4)	11.9 (4.5)	0.001
Fat tissue index (FTI) (kg/m^2^)	11.6 (6.7)	12.6 (7.8)	0.412	10.9 (5.4)	9.9 (5.5)	0.364
Lean tissue mass (LTM) (kg)	24.8 (7.4)	22.4 (9.1)	0.078	37.2 (10.3)	31.3 (10.5)	<0.001
Adipose tissue mass (ATM) (kg)	27.3 (16.1)	27.2 (18.7)	0.195	29.6 (14.9)	26.8 (13.1)	0.066
Total lipid mass (FAT) (kg)	20.1 (11.9)	20.0 (13.7)	0.187	21.8 (10.9)	19.6 (9.8)	0.052
Dry weight (kg)	55.2 (14.3)	51.6 (13.1)	0.058	68.3 (16.9)	61.0 (9.5)	<0.001
Hemoglobin (g/dL)	10.3 ± 1.4	9.8 ± 1.6	0.061	10.4 ± 1.5	9.7 ± 1.7	0.009
Albumin (g/dL)	3.8 (0.5)	3.6 (0.5)	0.080	3.7 (0.6)	3.4 (0.5)	<0.001
Blood urea nitrogen (mg/dL)	44.8 (48.5)	39.3 (35.4)	0.184	57.2 (43.7)	44.1 (39.2)	0.022
Serum creatinine (mg/dL)	6.9 (4.5)	4.4 (3.1)	0.003	8.5 ± 3.7	6.5 ± 3.4	0.001
Total cholesterol (mg/dL)	164.0 (64.0)	147.0 (46.2)	0.101	134.0 (50.5)	131.5 (40.2)	0.844
C-reactive protein (mg/L)	0.1 (0.5)	0.2 (0.7)	0.397	0.1 (0.7)	0.5 (1.0)	0.006
CONUT score	2.0 (3.0)	3.5 (2.2)	0.037	3.0 (2.0)	4.0 (2.8)	0.004

**Table 2:** Cox proportional hazards regression analysis of survival in dialysis patients and gender-based subgroups: subgroups according to lean tissue index (LTI), fat tissue index (FTI), body mass index (BMI), and handgrip strength (HGS) were defined using ROC analysis to evaluate the predictive value of these variables for all-cause mortality. Youden’s index was used to determine the optimal cut-off point for each variable. In the overall dialysis patient cohort, LTI, BMI, and HGS were significant predictors of survival. Gender-specific analysis revealed that in males, both LTI and HGS significantly predicted survival, whereas in females, HGS was the strongest predictor, with LTI also showing significance. HR: hazard ratio, CI: confidence interval.

	Lean Tissue Index		Fat Tissue Index		Body Mass Index		Handgrip Strength	
	HR (90% CI)	*p*-Value	HR (90% CI)	*p*-Value	HR (90% CI)	*p*-Value	HR (90% CI)	*p*-Value
Overall	3.30 (1.75, 6.19)	<0.001	1.55 (0.76, 3.18)	0.231	2.65 (1.17, 6.01)	0.020	4.22 (2.05, 8.70)	<0.001
Male	4.81 (1.89, 12.23)	<0.001	1.78 (0.68, 4.69)	0.242	2.45 (0.88, 6.81)	0.085	5.45 (2.18, 13.61)	<0.001
Female	3.22 (1.24, 8.40)	0.017	3.51 (0.92, 12.52)	0.053	4.57 (0.61, 34.19)	0.139	6.01 (2.42, 14.94)	<0.001

**Table 3:** Association of handgrip strength with mortality by Cox proportional hazards analysis in dialysis patients and gender-based subgroups using different models: Multivariate analysis using different models showed that in Model 2, the difference in survival rates based on HGS was statistically significant in all patients and in both gender groups. In Model 3, statistical significance was confirmed in the overall patient cohort and in female patients. Model 1: unadjusted; Model 2: adjusted for age, DM, HTN, BMI, FTI, and LTI; Model 3: adjusted for age, DM, HTN, BMI, FTI, LTI, serum albumin, and CONUT score. HR: hazard ratio, CI: confidence interval, BMI: body mass index, DM: diabetes mellitus, FTI: fat tissue index, HTN: hypertension, LTI: lean tissue index.

	Model 1 (Univariate)		Model 2 (+Age, DM, HTN, BMI, FTI, LTI)		Model 3 (Model 2 + Albumin, CRP, CONUT)	
	HR (90% CI)	*p*-Value	HR (90% CI)	*p*-Value	HR (90% CI)	*p*-Value
Overall	4.22 (2.05, 8.70)	<0.001	3.36 (1.54, 7.34)	0.002	2.86 (1.30, 6.28)	0.009
Male	5.45 (2.18, 13.61)	<0.001	4.48 (1.63, 12.28)	0.004	2.59 (0.83, 8.11)	0.102
Female	6.01 (2.42, 14.94)	<0.001	3.50 (1.28, 9.57)	0.015	2.77 (1.00, 7.65)	0.049

## Data Availability

The original contributions presented in the study are included in the article/Supplementary Material; further inquiries can be directed to the corresponding authors.

## Supplementary Materials

The following supporting information can be downloaded at: https://www.mdpi.com/article/10.3390/diagnostics15010075/s1, Figure S1: receiver operating characteristic (ROC) curves for predicting mortality using handgrip strength (HGS), lean tissue index (LTI), fat tissue index (FTI), and body mass index (BMI); Table S1: predictive performance of body composition and handgrip strength measurements for mortality in dialysis patients.
