# Supplementary material for: Impact of Handgrip Strength on Survival in Hemodialysis Patients
Source: Diagnostics (Basel). 2024 Dec 31;15(1):75. doi: 10.3390/diagnostics15010075 (PMC11719649; doi:10.3390/diagnostics15010075)
Supplement: Supplementary file 1 [file diagnostics-15-00075-s001.zip › diagnostics-3368763-supplementary.pdf]

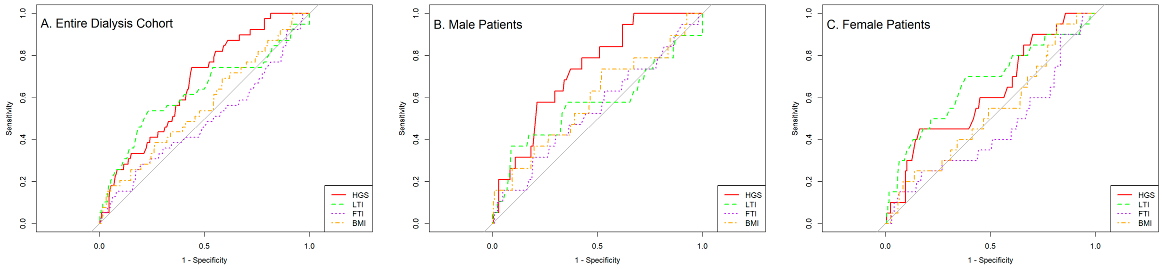

Figure S1. Receiver Operating Characteristic (ROC) curves for predicting mortality using handgrip strength (HGS), lean tissue index (LTI), fat tissue index (FTI), and body mass index (BMI). Panel A represents the entire cohort, Panel B focuses on male patients, and Panel C focuses on female patients.

Table S1. Predictive performance of body composition and handgrip strength measurements for mortality in dialysis patients : This table summarizes the area under the curve (AUC), 95% confidence intervals (CI), cut-off values, sensitivity, and specificity for handgrip strength (HGS) and body composition measurements (lean tissue index [LTI], fat tissue index [FTI], and body mass index [BMI]) in predicting mortality. Results are presented for the entire dialysis cohort, male patients, and female patients.

	Predictive index	AUC (95% CI)	Cut off value	Sensitivity (%)	Specificity (%)
Entire dialysis cohort	HGS	0.666 (0.584-0.747)	18.4	74.4	56.1
	LTI	0.626 (0.517-0.734)	9.95	53.8	76.7
	FTI	0.488 (0.382-0.593)	7.55	25.6	82.7
	BMI	0.562 (0.463-0.660)	18.9	17.9	94.6
Male patients	HGS	0.730 (0.626-0.834)	18.5	57.9	78.7
	LTI	0.571 (0.404-0.739)	9.6	36.8	91.1
	FTI	0.546 (0.400-0.692)	7.6	31.6	80.7
	BMI	0.591 (0.444-0.737)	25.3	73.7	48.0
Female patients	HGS	0.621 (0.489-0.754)	10.2	45.0	83.8
	LTI	0.667 (0.525-0.809)	9.95	70.0	61.7
	FTI	0.450 (0.304-0.595)	5.85	15.0	93.4
	BMI	0.529 (0.393-0.666)	27.9	95.0	18.6
